# The Effect of Recovery Duration on Vastus Lateralis Oxygenation, Heart Rate, Perceived Exertion and Time Motion Descriptors during Small Sided Football Games

**DOI:** 10.1371/journal.pone.0150201

**Published:** 2016-02-26

**Authors:** Scott McLean, Hugo Kerhervé, Geoff P. Lovell, Adam D. Gorman, Colin Solomon

**Affiliations:** 1 School of Health and Exercise Science, University of the Sunshine Coast, Sippy Downs, Australia; 2 School of Social Sciences, University of the Sunshine Coast, Sippy Downs, Australia; 3 Laboratoire de Physiologie de l’Exercice, Université de Lyon, F–42023, Saint–Etienne, France; Research Center for Sports Sciences, Health and Human Development (CIDESD), University of Trás-os-Montes e Alto Douro, Vila Real, Portugal, PORTUGAL

## Abstract

**Purpose:**

Small sided games (SSG) of football are an effective and efficient format to simultaneously train the physiological, technical, and tactical components of football. The duration of the recovery period between bouts of SSG will affect the physiological response to subsequent bouts. It was hypothesised that decreasing the duration of recovery periods separating serial SSG bouts would increase physiological, and perceptual responses, and decrease high speed running, and distance during SSG bouts.

**Methods:**

Twelve experienced footballers (mean ± SD; age 21 ± 3 yrs; VO_2peak_ 64 ± 7 ml·min·kg^−1^; playing experience 15 ± 3 yrs) completed two SSG sessions. Each SSG consisted of 3 vs. 3 players and 6 bouts of 2 min duration, with bouts separated by either 30 s recovery (REC-30) or 120 s recovery (REC-120). Deoxygenated haemoglobin (HHb) in the vastus lateralis (VL) (using near infrared spectroscopy), heart rate (HR) and time motion descriptors (TMD) (speed and distance) were measured continuously during the SSG sessions and perceived exertion (RPE) was measured for each bout.

**Results:**

During the recovery periods, in REC-30 compared to REC-120, there was a significant (p < 0.05) main effect of a higher HHb and HR. During the bouts, in REC-30 compared to REC-120, there were no significant differences in HHb, HR, RPE, or TMD, but within both REC-30 and REC-120 there were significant increases as a function of bout number in RPE.

**Conclusions:**

Although a four-fold increase in recovery period allowed a significant increase in the recovery of HHb and HR, this did not increase the physiological, and perceptual responses, or time motion descriptors during the bouts. These results could have been due to the regulation of effort (pacing), in these experienced players performing an exercise task to which they were well adapted.

## Introduction

Small sided games (SSG) are a form of football training used to simultaneously train the physiological, technical, and tactical components of football [1–3]. Typically, SSG are used in an interval training format, consisting of a series of bouts and recovery periods [4–7]. A commonly used SSG format is played with 3 vs 3 players [6–9], in which, during the bouts, there are average heart rate (HR) responses of higher than 80% of maximal HR values (%HR_max_), blood lactate concentration [La] values ranging from 5 mmol^-1^ to 8.5 mmol^-1^, and ratings of perceived exertion (RPE) of 8.5 (CR-10) and 16.3 (Borg 6–20) [6,8,10–13]. In addition to the physiological and perceptual measures of exercise intensity during SSG, time motion descriptors (TMD) (speed and distance) achieved by players, measured by global positioning systems (GPS) permits estimations of external exercise intensity [5,14–16]. The high levels of these direct and indirect measures of exercise intensity, indicate that SSG bouts are an effective training stimulus for increasing the aerobic power of football players [17,18].

Several modifiable variables of SSG are known to influence the exercise intensity of SSG [3]. Decreasing the players per team [14,19,20], increasing the pitch size [4,6,21], decreasing the amount of touches per individual possession [5], including verbal coach encouragement [6], and decreasing bout duration [22] all increase the exercise intensity of SSG. These modifiable SSG variables allow SSG to be adapted to train specific components of football.

However, only one study has investigated the effect of different recovery durations between serial bouts on physiological, perceptual, time motion and technical variables during SSG [9]. Using inexperienced players, the SSG format consisted of 3 vs 3 players per team with recovery durations of 1 min, 2 min, 3 min and 4 min separating 4 x 4 min SSG. The shorter recovery durations (1 and 2 min.), compared to longer recovery durations (3 and 4 min), induced higher HR and blood lactate values, and participants covered more distance in the lower speed zones. There was no difference in the participants RPE between the conditions.

The ability to maintain a high exercise intensity across multiple exercise bouts is dependent on the recovery from the previous exercise bouts [23]. This is influenced by both the exercise intensity during the bouts, and the duration of the recovery periods [24]. During recovery from exercise, oxygen (O_2_) consumption remains elevated to replenish the intramuscular high energy phosphates required to perform high intensity exercise, to pre-exercise levels [23–25]. As football involves brief (2–4 s), frequent periods of maximal intensity exercise during a match [26–28], the subsequent recovery duration from these efforts may be insufficient to fully resynthesise the intramuscular high energy phosphates [23,29]. Progressive depletion of adenosine triphosphates (ATP) and phosphocreatine (PCr) stores increases the reliance on energy provided from anaerobic glycolysis, resulting in increased H^+^ and reduced pH levels [24,30]. This increase in anaerobic energy transfer is associated with decrements in repeat sprint times, accelerations, mean running speed, and power output across subsequent exercise bouts [23,30]. Decreased oxygen availability is expected to be the limiting factor of ATP and PCr resynthesis during the initial period (up to 30 s) of recovery from exercise [25,31]. This indicates the importance of O_2_ availability and utilisation for recovery from exercise. Therefore, the assessment of specific muscle tissue oxygenation is necessary to understand local muscle metabolism during football specific exercise.

Near infrared spectroscopy (NIRS) allows portable and non-invasive investigation of specific regional tissue oxygenation changes during exercise [32]. Measurements of oxygenated haemoglobin (O_2_Hb), deoxygenated haemoglobin (HHb) and total haemoglobin (tHb) are used to determine the balance between oxygen (O_2_) supply and utilisation at the site of investigation [33,34]. During exercise that utilises aerobic energy transfer there is an increased demand for O_2_ in the active muscles, resulting in increased HHb and decreased O_2_Hb [33]. However, during recovery from exercise this balance is reversed, due to decreased extraction of oxygen at the tissue [33]. Regional muscle tissue oxygenation has been assessed during repeat sprint running, and cycling [35–37]. Despite the exercise mode, there was a consistent increase in [HHb] during exercise, and decreased [HHb] during recovery periods [35,36].

There is currently no research specific to local muscle oxygenation changes during football specific exercise such as SSG, and the effect of different recovery durations. During running, active recovery, compared to passive recovery of the same duration, separating repeated sprints elicited higher [HHb] in the vastus lateralis (VL) [36]. The higher [HHb] was associated with increased repeated sprint times during subsequent sprints [36]. During repeat cycling sprints, [O_2_Hb] increased and [HHb] decreased with longer recovery durations, despite whether active or passive recovery was utilised [37]. This increase in O_2_Hb was associated with reduced decrements in percentage of peak power outputs across subsequent sprints [37]. Therefore, when using SSG as interval training it is necessary to control the duration of the recovery periods separating bouts, so that the desired training effect is achieved.

The aim of this study was to determine the effect of increasing the recovery duration in a 3 vs 3 player SSG format on VL oxygen utilisation, HR, RPE and TMD in experienced footballers. It was hypothesised that: 1) During the SSG recovery periods, a 120 s recovery duration compared to a 30 s recovery duration, would decrease VL HHb concentrations and HR. 2) During the serial SSG bouts, a 120 s recovery period compared to a 30 s recovery period, would decrease VL HHb, HR and RPE, and players would cover more total distance and spend more time in high speed zones.

## Methods

### Ethical Approval

This study was approved by the Human Research Ethics Committee at the University of the Sunshine Coast (HREC: S/14/661), and participants provided written informed consent.

### Participants

Twelve experienced male footballers (mean ± SD; age 21.3 ± 2.9 years; weight 75.0 ± 7.1 kg; height 179.5 ± 6.9 cm; HR_peak_ 190 ± 10.2 beats·min^−1^; VO_2peak_ 64 ± 7.4 ml·min·kg^−1^; playing experience 14.6 ± 3.1 years) of the same team, playing in the second tier of Australian football participated in the study. Participants trained three times per week for an approximate weekly total of 240 min and competed in one match per week of 90 min duration. Inclusion in the study required the successful completion of a medical health questionnaire (PAR-Q).

### Project design

The study was conducted using a one-group, crossover, counterbalanced, repeated measures design. The independent variables were the two different recovery durations, 30 s (REC-30), 120 s (REC-120), and the number of bouts (1–6). The dependent variables measured were oxygenated haemoglobin (O_2_Hb), deoxygenated haemoglobin (HHb), total haemoglobin (tHb), heart rate (HR), rating of perceived exertion (RPE) (Borg CR-10) and time motion descriptors (TMD) (speed and distance). Participants completed the SSG sessions, a peak aerobic capacity test in laboratory conditions, and a test of maximal running speed over 20 m. All testing and data collection was completed in an 11 week period during the participant’s competitive season. One 3-a-side team was equipped with the NIRS devices (one device for each player) for each SSG, and only data from these participants were used for this study.

### Small Sided Games

Each participant completed REC-30 and REC-120 under the same SSG format consisting of 3 vs 3 players with 6 x 2 min bouts played on a 15 m x 20 m natural grass pitch. Each participant was tested in two separate session, separated by a minimum of two days and maximum of five days, and the order of conditions was counter-balanced. The SSG testing was completed in eight sessions over five weeks. The SSG testing sessions were completed at the beginning of the participant’s normal football training session and at the same time of day to avoid variations to circadian rhythm. The SSG teams were selected by the two experienced team coaches to ensure that similar levels of technical ability and physical capacity of players were evenly distributed throughout the teams. The same team membership was used for all testing sessions. The objective of the SSG was to maintain possession as a team, with unlimited ball contacts per possession, no goals and goalkeepers and no coach encouragement. Additional balls were placed around the pitch to minimise time lost for balls out of play. Prior to testing participants performed a standardised warm up. Immediately post warm up the participants were fitted with NIRS, HR straps and GPS devices before beginning the SSG. During the recovery periods participants were instructed that they could walk within the playing grid after their RPE was recorded.

### Oxygenation in Vastus Lateralis Muscle

Oxygenation in the VL was assessed by portable wireless (Bluetooth) NIRS devices (Portamon, Artinis, Medical system, Zetten, The Netherlands) using a two wave length continuous wave system. The relative changes in HHb, O_2_Hb, and tHb were measured at 10 Hz using the differences in absorption of light at 750 and 850 nm. Due to the uncertainty of the proton path in biological tissue, a fixed differential path length correction factor was applied as recommended by the manufacturer. The NIRS measurements were conducted on the VL of the left leg. The VL was the chosen site because of its large contribution to running, directional changes, and kicking in football [38,39]. The location of the NIRS device was one third of the distance from the lateral femoral epicondyle and the greater trochanter of the femur and adjusted medially to be positioned on the belly of the VL. This position was recorded for each participant at the first testing session and reproduced in the subsequent sessions. The NIRS device was fixed to the skin of the participant by elastic adhesive bandage. Skinfold thickness at the site of measurement was measured (British Indicators Ltd, Burgess Hill UK) to ensure optimal illumination of the muscle tissue (< 17 mm was required in this study).

Analysis of HHb was conducted because it is relatively independent of blood volume changes during exercise [35,36]. A moving average filter width of 1 s was applied to the raw data to reduce high frequency noise before exporting. A 5 s moving average filter was applied to the data to minimise variability in the individual data, before calculating the average for the total duration of each bout and recovery periods to allow for the determination of the pattern of response over time. The averaged HHb of REC-30 and REC-120 was compared between conditions, for the bouts and recovery periods. The NIRS data is the relative change in concentration from baseline and is expressed in micromolar units (μM). Baseline was the average of the 30 s (standing rest inside the playing area), measured immediately prior to commencement of the first bout of each testing session. To determine the between test reliability of the HHb data from the NIRS system, absolute (Typical Error 0.92), and relative (Intra-class Correlation .702) reliability of the baseline data were used [40]. Previous research has shown that the NIRS method provides acceptable reliability [41].

### Heart Rate

During the SSG, HR was recorded continuously (Polar strap and Catapult MinimaxX S4 recorder; Catapult Sports, Melbourne). The HR responses of participant recorded during the SSG were averaged for the bout and recovery periods and compared between conditions. The HR is expressed as a beats per minute (beats·min^−1^). HR data was n = 8 for REC-30, and n = 9 for REC-120.

### Rating of Perceived Exertion

During the first 10–15 s of each recovery period, participants RPE (Borg CR-10) response was recorded to measure the global perceived effort (intensity) of the previous bout of SSG. Participants were familiarised with the RPE scale during normal training sessions in the two weeks leading up to the testing sessions.

### Time Motion Descriptors

The distance covered and speed attained by participants during the SSG was measured at a 10 Hz sampling rate by portable GPS units (MinimaXS4, Catapult sports, Melbourne), and analysed in Catapult Sprint 5.01 software. The GPS devices were worn in a harness on the upper back. The total distance covered in the SSG session, distance covered in each bout, and percentage of time spent in four speed zones 0–6.9 km·h^−1^ (0–1.9 *m·s*^−1^) 7–12.9 km·h^−1^ (1.9–3.6 *m·s*^−1^), 13–17.9 km·h^−1^ (3.6–5.0 *m·s*^−1^), > 18 km·h^−1^ (> 5.0 *m·s*^−1^) were analysed [9].

### Peak Exercise Test

An incremental intensity exercise test to volitional cessation was conducted to obtain the participants peak aerobic capacity using a treadmill (T200, Cosmed, Rome Italy). During the peak exercise test, expired ventilation and oxygen and carbon dioxide utilisation, carbon dioxide production and the respiratory exchange ratio were measured, using a two-way, non-rebreathing valve (series 2700, Hans-Rudolf, Kansas City, USA) and an automated open-circuit spirometry metabolic analysis system (True One 2400, Parvo Medics, Sandy UT). Stages 1–3 of the continual peak exercise test were conducted at 1% incline (6 km·h^−1^ for 3 mins, 8 km·h^−1^ for 1 min, and 10 km·h^−1^ for 1 min). For stages 3 onward speed was constant at 10 km·h^−1^ and gradient increased by 2% each min until volitional cessation.

The maximal exercise test was used to obtain the maximal HR of participants. A polar heart rate strap and open-circuit spirometry metabolic analysis system (True One 2400, Parvo Medics, Sandy UT) recorded continuous HR via short range telemetry. The RPE (Borg CR-10) was recorded during the last 20 s of each one minute stage, beginning at 5% incline until completion of the test.

### Peak Speed Test

During pilot testing, some unrealistically high speed values from the GPS data were identified, possibly because of GPS sampling error, or the inaccuracy of GPS during changes of direction at high speed [42]. To set a realistic maximum limit for the speed data from the GPS, the maximal running speed achievable by participants within the playing area (15 m x 20 m) was determined. Participants performed three maximal sprints of 20 m through a series of electronic timing gates (Smartspeed, Fusion Sports). The group average of the participant’s fastest trials, with an added 5% was then used to set an upper limit of 34.4 km·h^−1^(9.5 *m·s*^−1^) for the GPS velocity data analysis. Any data above this speed threshold was removed prior to analysis.

### Statistical Analysis

The data analysed in this study was only from participants that were wearing the NIRS devices during the SSG. Sample size calculations (in G*Power) using the mean difference (5.5 μM) and standard deviation (4.7 μM) in HHb from baseline to exercise in a comparable study [35], and using a power of 0.80 and probability of 0.05, indicated that the twelve participants of the current study would provide sufficient statistical power. Statistical analysis were performed using SPSS (version 22, IBM Corporation, Armonk NY, USA). Data are reported using mean ± SD, and the level of significance was set at p < .05. A two-way repeated-measures ANOVA was used to assess the effect of condition (REC-120 and REC-30), bout (1–6), and condition x bout interactions for all dependent variables. Assumptions of sphericity using Maulchy’s W test was used, and in all cases where Maulchy’s W was significant, the Greenhouse-Geisser correction of degrees of freedom was used. Post hoc pairwise comparisons were used to follow-up significant interactions, and were conducted using the least significant difference (LSD) test with no adjustments for multiple comparisons. Calculations of p value, partial eta-squared (as an indicator of effect size, noted ɳ_Ƥ_^2^) and statistical power (ß) were reported.

## Results

### Vastus Lateralis Oxygenation

**Fig 1:**
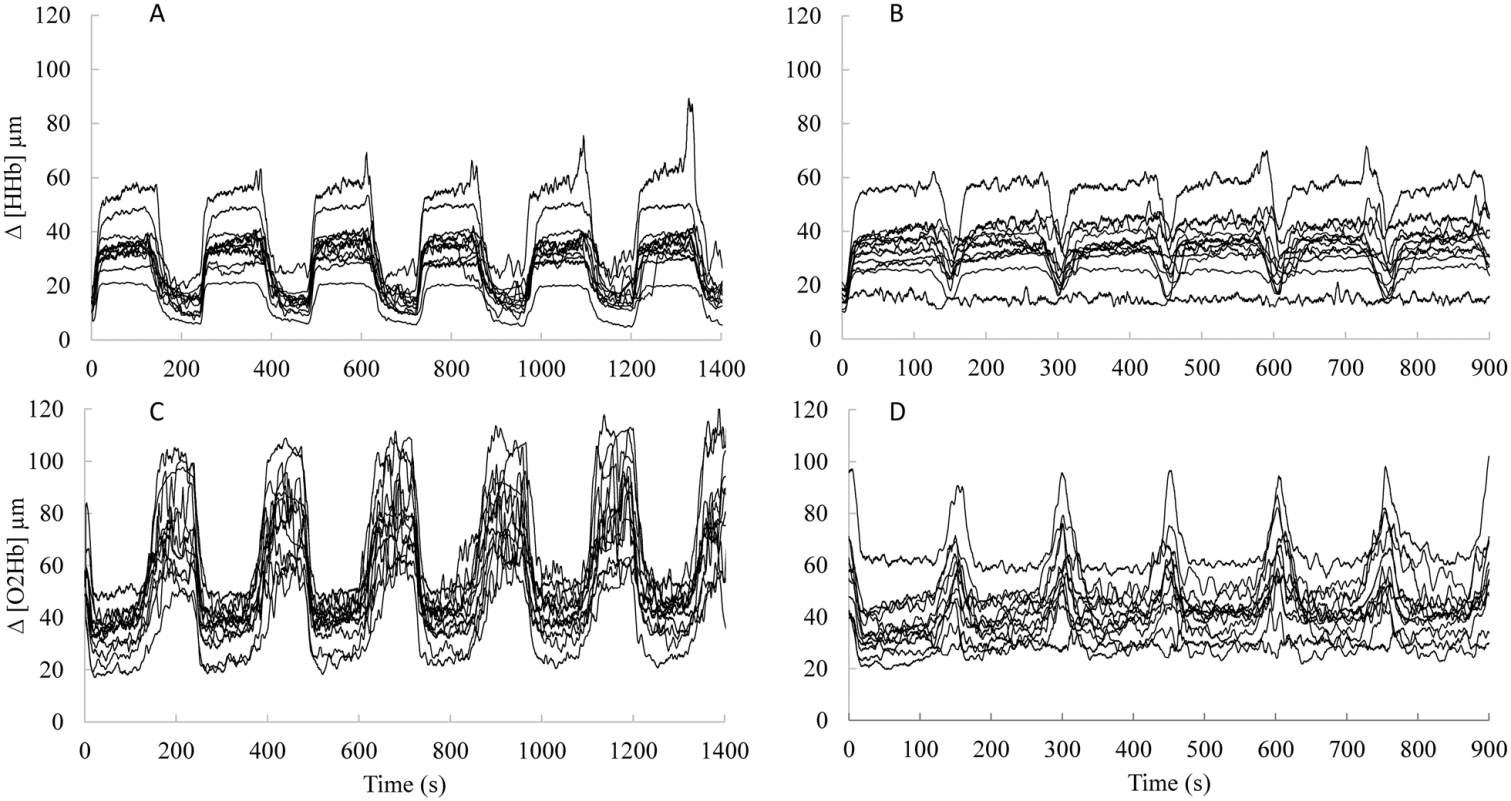
Individual oxygenation changes during the SSG sessions. Panel (A) Δ [HHb] during REC-120. Panel (B) Δ [HHb] during REC-30. Panel (C) Δ [O_2_Hb] during REC-120. Panel (D) Δ [O_2_Hb] for REC-30.

Individual oxygenation changes during the SSG sessions (Fig 1). The VL oxygenation followed the expected pattern of change of increased HHb and decreased O_2_Hb during the bouts, and increased O_2_Hb and decreased HHb during the recovery, with the associated intra-individual and inter-individual variability (Fig 1).

### Deoxygenated Haemoglobin (HHb)

#### Recovery

**Fig 2:**
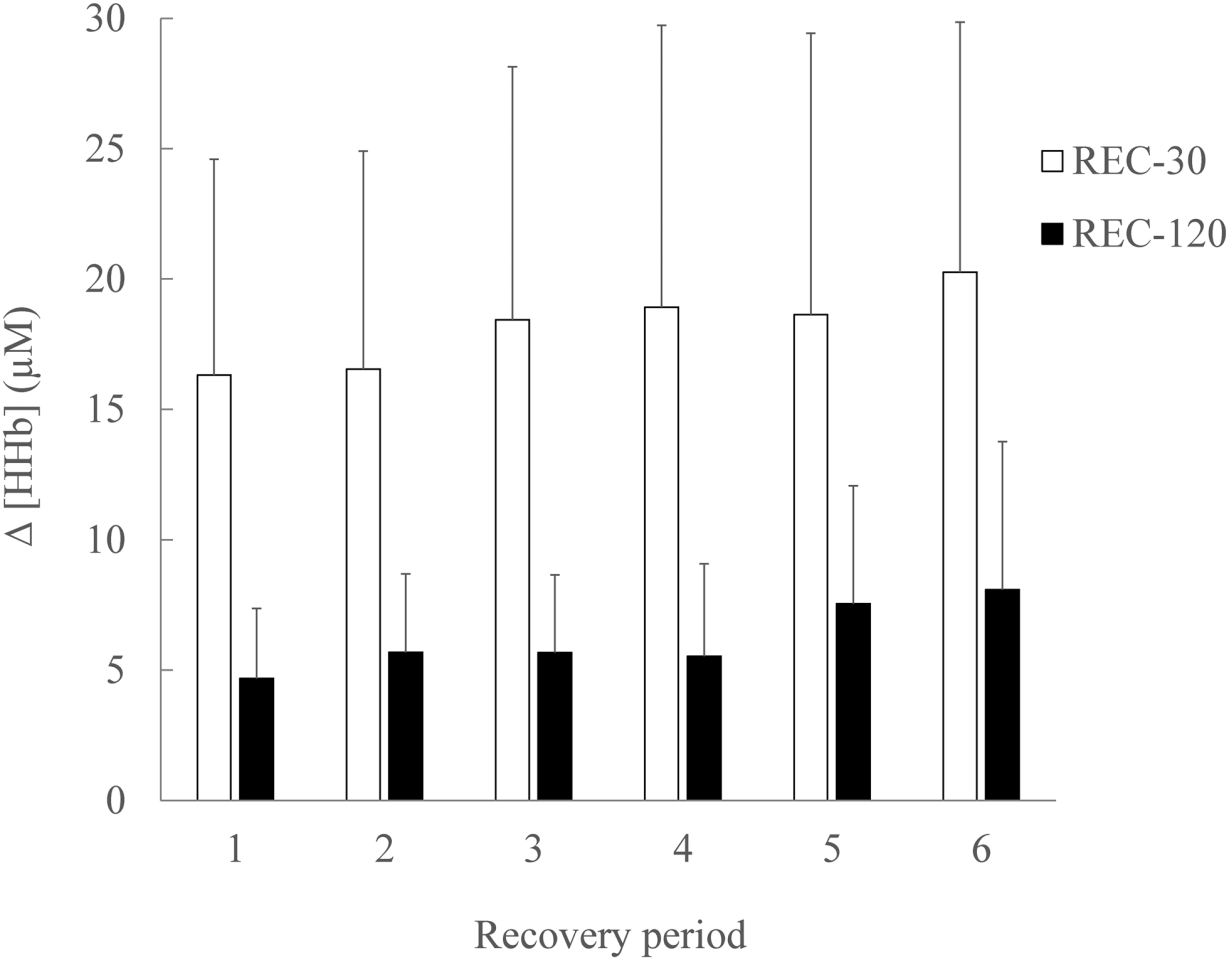
Relative change from baseline of vastus lateralis (VL) muscle deoxygenated haemoglobin (HHb) concentration during SSG recovery periods. There was a significant main effect (p < .05) of condition, and bout number, with an increase of the average HHb. There was no significant (p > .05) condition x bout interaction for the average HHb. Data are mean ± SD.

For HHb there was a significant main effect of condition (p <0.001; ɳ_Ƥ_^2^ = 0.725; ß = 0.998), and bout number (p = 0.001; ɳ_Ƥ_^2^ = 0.457; ß = 0.965), with an increase of the average HHb during the recovery periods (Fig 2). There was no significant condition x bout interaction for the average HHb (p = 0.295; ɳ_Ƥ_^2^ = 0.105; ß = 0.302) during the recovery periods (Fig 2).

#### Bouts

**Fig 3:**
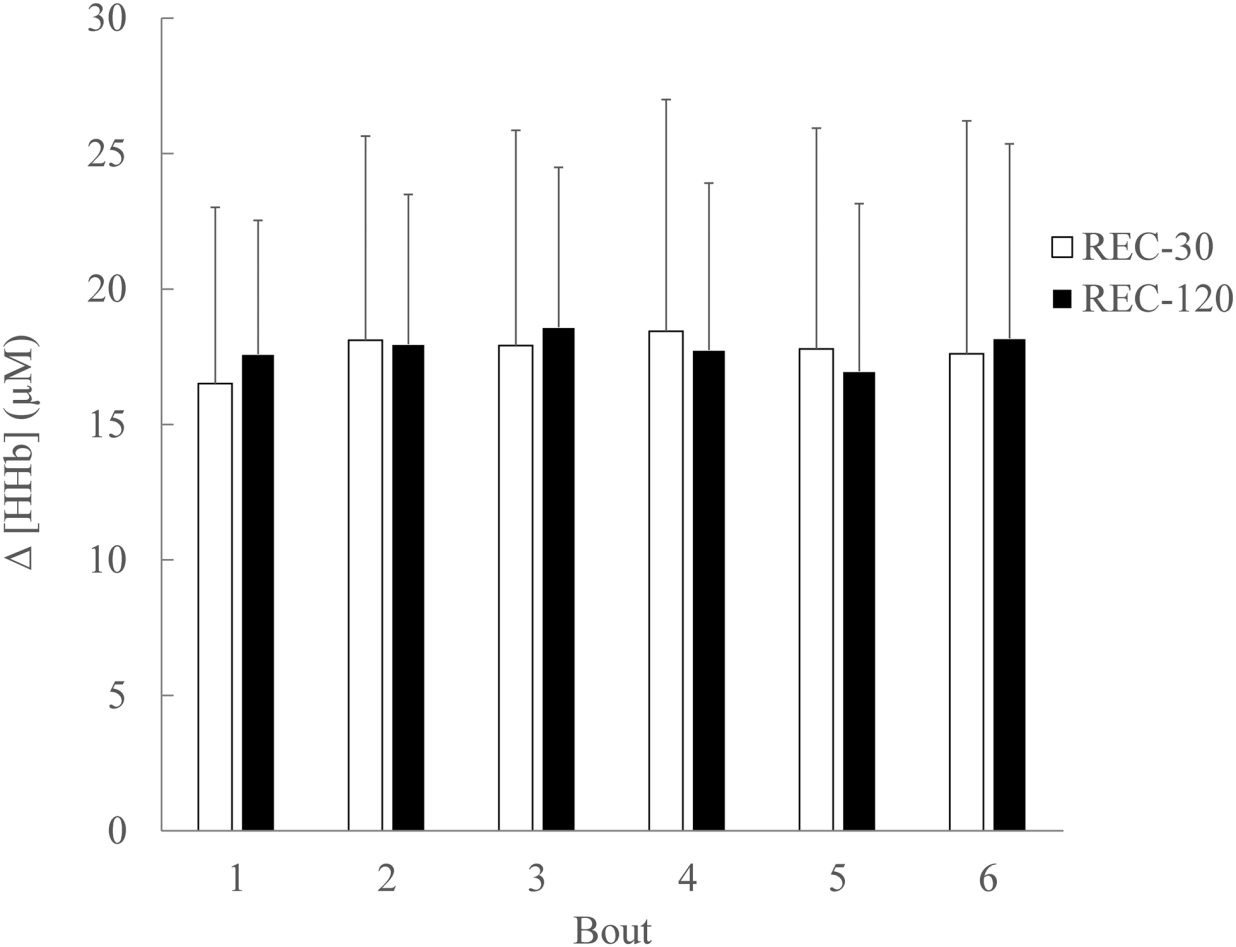
Relative change from baseline of vastus lateralis (VL) muscle deoxygenated haemoglobin (HHb) concentration during SSG bouts. There was no significant main effect (p > .05) of condition, or bout number for the average HHb. There was no significant (p > .05) condition x bout interaction for the average HHb. Data are mean ± SD.

For HHb there was no significant main effect of condition (p = 0.935; ɳ_Ƥ_^2^ = 0.000; ß = 0.050), or bout number (p = 0.231; ɳ_Ƥ_^2^ = 0.126; ß = 0.284) for the average HHb during the bouts (Fig 3). There was no significant condition x bout interaction for the average HHb (p = 0.306; ɳ_Ƥ_^2^ = 0.101; ß = 0.404) during the bouts (Fig 3).

### Heart Rate (HR)

#### Recovery

**Fig 4:**
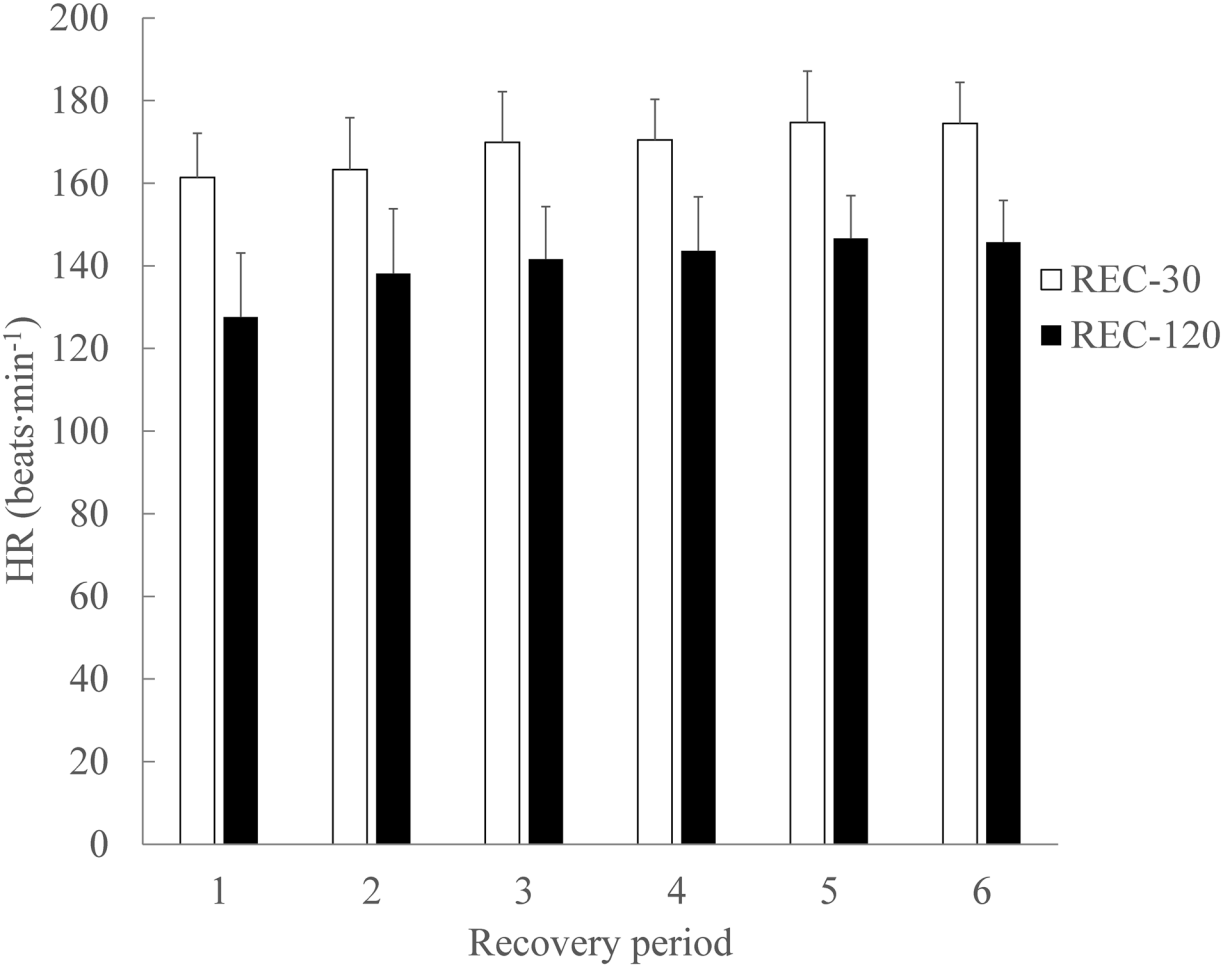
Average heart rate (beats·min^−1^) during SSG recovery periods. There were significant main effects (p < .05) of condition, and bout number, with an increase of the average HR (beats·min^−1^). There was no significant (p > .05 condition x bout interaction for the average HR (beats·min^−1^). Data are mean ± SD.

For HR there was a significant main effect of condition (p = 0.001; ɳ_Ƥ_^2^ = 0.849; ß = 0.997) with an increased HR in REC-30, and bout number (p <0.001; ɳ_Ƥ_^2^ = 0.838; ß = 1.000), during the recovery periods (Fig 4). There was no significant condition x bout interaction for the average HR (beats·min^−1^) (p = 0.591; ɳ_Ƥ_^2^ = 0.111; ß = 0.233) during the recovery periods (Fig 4).

#### Bouts

**Fig 5:**
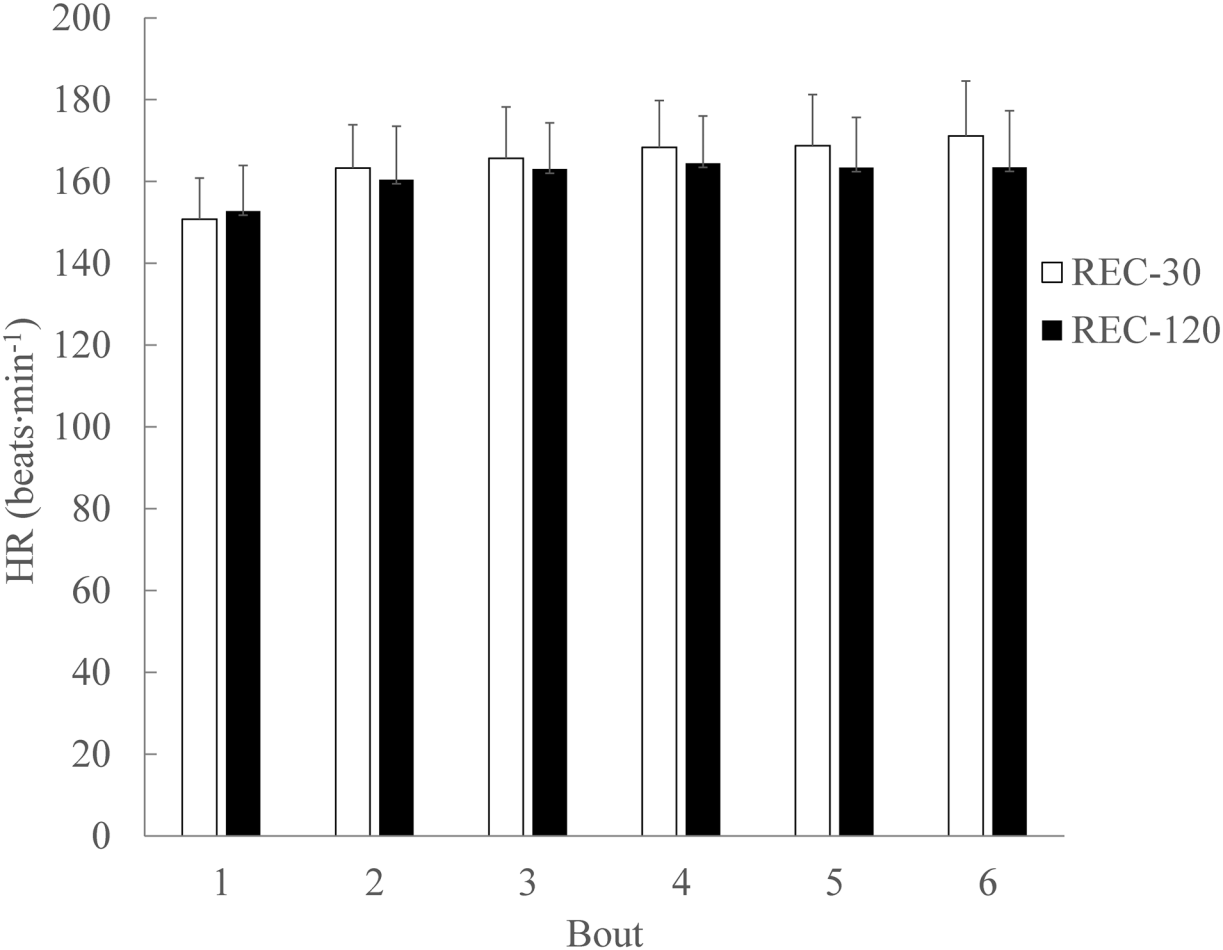
Average heart rate (beats·min^−1^) during SSG bouts. There was no significant main effect (p > .05) of condition for average the HR (beats·min^−1^). There was a significant main effect (p < .05) of bout number, with an increase of the average HR (beats·min^−1^) across the bouts. There was no significant condition x bout interaction (p > .05) for the average HR (beats·min^−1^). Data are mean ± SD.

For HR there was no significant main effect of condition for the average HR (beats·min^−1^) (p = 0.295; ɳ_Ƥ_^2^ = 0.180; ß = 0.163) during the bouts (Fig 5). There was a significant main effect of bout number, with an increase of the average HR (beats·min^−1^) (p <0.001; ɳ_Ƥ_^2^ = 0.883; ß = 1.000) across the bouts (Fig 5). There was no significant condition x bout interaction for the average HR (beats·min^−1^) (p = 0.133; ɳ_Ƥ_^2^ = 0.236; ß = 0.548) during the bouts (Fig 5).

### Rating of perceived exertion (RPE)

**Fig 6:**
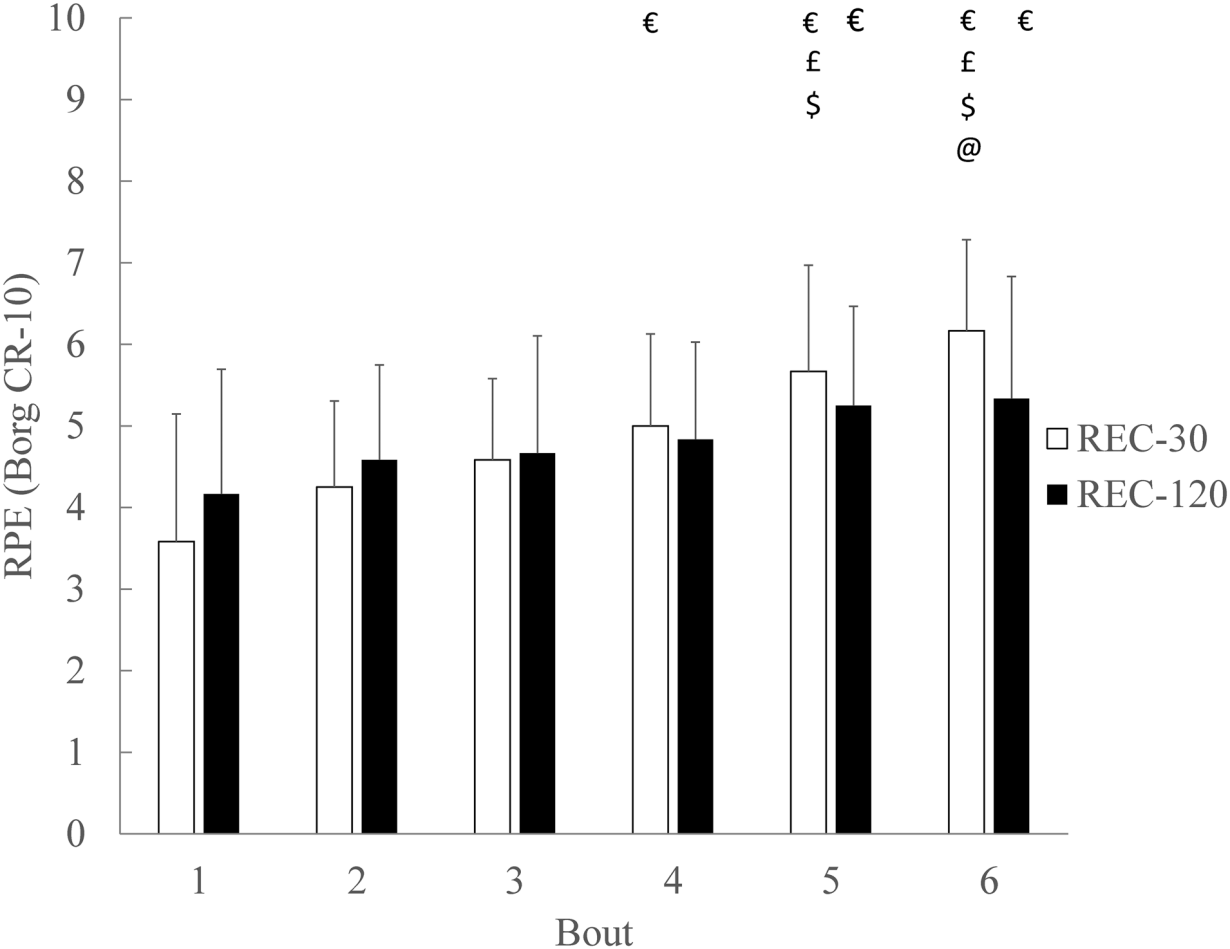
Rating of perceived exertion (RPE) during SSG bouts. There was no significant main effect (p > .05) of condition on RPE. There was a significant main effect (p < .05) of bout number, with an increase in RPE across the bouts. There was a significant (p < .05) condition x bout interaction for RPE. Data are mean ± SD. Symbols denote significant differences from post hoc pairwise comparisons (€) to B1, (£) to B2, ($) to B3 and (@) to B4.

For RPE there was no significant main effect of condition (p = 0.824; ɳ_Ƥ_^2^ = 0.005; ß = 0.055) during the bouts (Fig 6). There was a significant main effect of an increase across the bouts (p < 0.001; ɳ_Ƥ_^2^ = 0.610; ß = 1.000). For RPE there was a significant condition x bout interaction (p = 0.016; ɳ_Ƥ_^2^ = 0.218; ß = 0.836) (Fig 6).

The post hoc pairwise comparisons indicate that in REC-120 RPE increased from B1 to B5 (p < 0.048), and B6 (p = 0.033). In REC-30 RPE increased from B1 to B4 (p = 0.008), B5 (p< 0.001) and B6 (p < 0.001), from B2 to B5 (p = 0.008) and B6 (p < 0.001), from B3 to B5 (p = 0.040) and B6 (p = 0.003), and from B4 to B6 (p = 0.027) (Fig 6). There were no significant (p > 0.05) differences between the conditions for each bout.

### Time motion descriptors

#### Total distance covered during the SSG session

For total distance covered during the SSG session there was no significant (p = 0.638) difference between REC-30 (1365 ± 37.7 m) and REC-120 (1347 ± 37.7 m).

#### Distance covered during the SSG bouts

**Fig 7:**
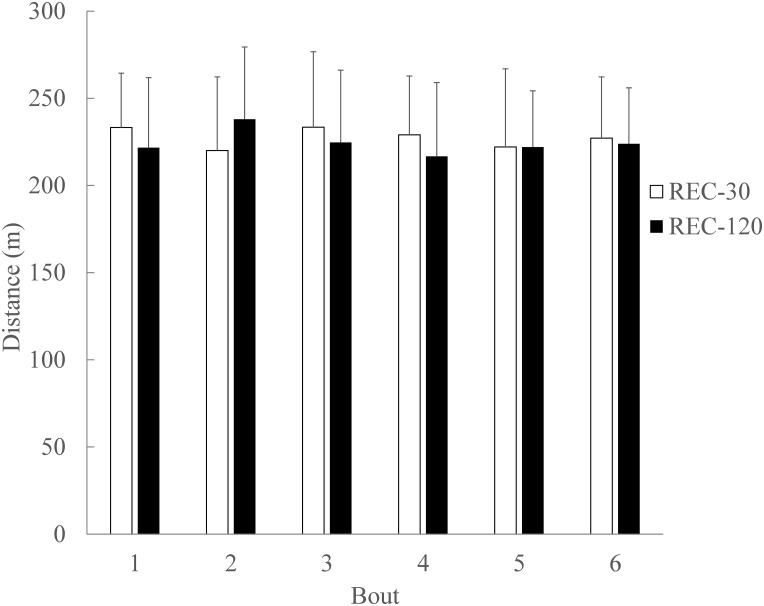
Total distance covered during SSG bouts. There was no significant main effect (p > .05) of condition, or bout number for total distance covered during the bouts. There was no significant (p > .05) condition x bout interaction for total distance covered during the bouts. Data are mean ± SD.

For total distance covered during the bouts there was no significant main effect of condition (p = 0.795; ɳ_Ƥ_^2^ = 0.006; ß = 0.057), or bout number (p = 0.914; ɳ_Ƥ_^2^ = 0.026; ß = 0.117) (Fig 7). There was no significant condition x bout interaction for total distance covered during the bouts (p = 0.515; ɳ_Ƥ_^2^ = 0.072; ß = 0.284) (Fig 7). Data are mean ± SD.

#### Percentage of time spent in speed zones during SSG bouts

There was no significant (p > 0.05) main effect of condition, or bout number for percentage of time spent in all speed zones. There was no significant (p > 0.05) condition x bout interaction of bout number for percentage of time spent in all speed zones (Table 1).

**Table 1:** Percentage of time spent in speed zones.

	Speed zone km·h^−1^(m·s^−1^)	B1(%)	B2(%)	B3(%)	B4(%)	B5(%)	B6(%)
REC-30	0–6.9(0–1.9)	60 ± 14	66 ± 7	63 ± 9	64 ± 9	64 ± 12	64 ± 9
	7–12.9(1.9–3.6)	36 ± 13	31 ± 6	32 ± 9	32 ± 9	31 ± 10	31 ± 8
	13–17.9(3.6–5.0)	3 ± 2	3 ± 2	5 ± 3	3 ± 2	4 ± 3	4 ± 3
	>18(> 5.0)	1 ± 1	0 ± 0	1 ± 2	1 ± 2	0 ± 0	1 ± 2
REC-120	0–6.9(0–1.9)	62 ± 10	58 ± 12	63 ± 9	63 ± 11	62 ± 9	61 ± 8
	7–12.9(1.9–3.6)	34 ± 8	36 ± 9	33 ± 8	33 ± 8	34 ± 7	35 ± 8
	13–17.9(3.6–5.0)	4 ± 3	6 ± 4	4 ± 2	4 ± 4	4 ± 2	3 ± 2
	>18(> 5.0)	0 ± 1	0 ± 1	0 ± 0	0 ± 1	0 ± 1	0 ± 1

There was no significant (p > .05) main effect of condition, or bout number for percentage of time spent in all speed zones. Data are mean ± SD.

## Discussion

This study was designed to determine the effect of two different recovery durations, separating six, 2 min bouts of a 3 vs 3 player SSG on physiological, and perceptual responses, and speed and distance. The two main results were that: 1) increasing the recovery duration from 30 s to 120 s between serial SSG bouts allowed [HHb], and HR to significantly decrease during the recovery periods, and 2) despite this increased physiological recovery, there was no significant difference between conditions for [HHb], HR, RPE, or running speed, and distance, between REC-30 and REC-120, during the bouts.

### Oxygenation in Vastus Lateralis

The primary and unique dependent variable for this study was the HHb measured in the VL. The results of the study supported the hypothesis that during the REC-30 recovery periods, [HHb] would remain significantly higher than during the REC-120 recovery periods. In repeat sprint cycling, increasing the recovery duration from 25 s to 100 s between ten, 5 s maximal effort cycling sprints increased [O_2_Hb] and decreased [HHb] [37]. However, contrary to our hypothesis of a higher [HHb] in REC-30 compared to REC-120, was the absence of a significant difference in [HHb] between REC-30 and REC-120, during the bouts.

### Heart Rate

A secondary and expected finding of this study was that during the recovery periods, HR significantly decreased as recovery duration was increased from 30 s to 120 s. Also, the average HR measured during the recovery period increased in both conditions, but because there was no significant condition x bout interaction, pairwise comparisons were not conducted. One study using four SSG bouts measured a significant decrease in HR of approximately 10 beats·min^−1^ during the final recovery period [4]. However, during running and cycling interval protocols, an upwards drift in the HR response continued across subsequent recovery periods were identified [43,44].

During the bouts, there was no significant main effect of condition on the average HR. The participants maintained a HR of approximately 80% to 90% of maximal HR values throughout the SSG sessions. The HR values measured during the bouts in the current study are comparable to HR values from previous SSG studies [6,19]. However, the HR results of the current study are contrary to those in the only other study that has investigated the effect of different recovery durations separating serial SSG bouts on physiological responses [9]. Koklu et al. [9] reported that a recovery duration of 1 min compared to 3 min and 4 min, induced a significantly higher average HR during SSG exercise bouts. The contrasting results could be attributed to a difference in SSG formats, or playing experience of the participants, as it is know that experienced athletes are able to regulate effort during exercise compared to less experienced athletes [45,46]. Similar HR responses to those in the current study were measured in experienced runners during self-paced treadmill running (6 x 4 min bouts) separated by either a 1 min, 2 min, or 4 min recovery duration, where no difference was measured in the HR between the three different recovery conditions [43].

### Perceived Exertion

The RPE results of this study did not support our hypothesis that participants would have a higher perceived effort during bouts subsequent to a 30 s compared to a 120 s recovery duration. The current RPE results are supported by there being no significant difference between the 1 min, 2 min, 3 min, and 4 min recovery periods separating SSG bouts [9]. In both the current study, and one other [9], RPE ranged from “hard” to between “hard” and “very hard”. However, the RPE in Koklu et al. [9] was measured two minutes after the final 4 min bout. To provide more detail of the psychobiological response of players when subjected to different recovery durations separating SSG, a bout by bout analysis of RPE was performed in the current study. The significant increase of RPE across the bouts in this study, and in a 4 vs. 4 SSG format, with constant bout (4 x 4 min) and recovery (3 min) durations [5], reflects the increasing perception of effort across serial bouts.

### Speed and Distance

The current time motion results did not support our hypothesis that participants in REC-30 compared to REC-120, would cover less distance and spend less time in the high speed zones. Instead, no difference was measured for total distances covered, or time in all four speed zones, between the two conditions. These results indicate that the intensity and work performed between conditions was matched. The speed and distance results of the current study cannot be directly compared to those of other SSG studies because of differences in the methods, such as bout duration, pitch size, and player number [1,14,16]. However, in a study that altered the work: rest ratio during touch football SSG, a decrease in the work: rest ratio (more frequent recovery periods) was associated with increases in moderate, to very high-speed movements [47]. The current results indicate that the participants were able to regulate their running speeds and distances in order to complete the SSG session without compromising physical performance.

### Combined responses

The HHb and HR results in this study indicate that the SSG bouts, within and between conditions, were performed at a similar exercise intensity, as a higher exercise intensity would have increased both HR and [HHb] [48]. The TMD results indicate that the total amount of work performed, and time spent in specific speed zones was similar, within and between conditions. An explanation for these unexpected results for HR, HHb, and TMD could be that the participants regulated their effort (paced), to delay the occurrence of fatigue [49], to complete the SSG sessions without compromising performance [50–52].

It is accepted that athletes use pacing strategies during continuous exercise such as long distance running, and cycling [52,53]. However, the unpredictability of high intensity intermittent team sports makes pacing more difficult to implement [50,51,54]. Nevertheless, when implemented, the pacing strategies used by athletes in team sports are influenced by prior knowledge of the exercise duration, playing experience, playing standard, and physical fitness [50,51,54]. Deceiving participants of the duration of exercise provides evidence that the knowledge of the exercise duration influences pacing [49,50]. Greater amounts of high speed running is performed when a shorter duration of exercise in anticipated, and a greater amount of low speed activity is seen when the exercise duration is unknown [50]. These findings indicate that athletes preserve energy for any unexpected and unpredictable events [54]. In the unpredictable environment of team sports, this energy preservation allows for exercise intensity to be up or down regulated depending on the match status [54]. In the current study the participants were informed of the exercise duration, which would have allowed a conscious decision to be made for the appropriate exercise intensity to complete the SSG session, while maintaining performance. This decision, of these experienced participant’s, would likely have been based on prior knowledge of the task [53], as SSG were a regular component of their training.

The high level of exercise capacity, and playing standard of the current participants may explain the RPE results. The RPE continued to increase across the bouts, despite a levelling off of HR and HHb, and with no change in the TMD across the bouts. These results indicate that although participants perceived the work to harder towards the end bouts, they were able to tolerate the workload and maintain the exercise intensity. It has been shown that players of a high playing standard and exercise capacity, pace at high levels of exercise intensity [51]. This phenomenon was indicated in the current study as HR was maintained at 80 to 90% HR_max_, across the bouts in both conditions.

## Conclusion

It is concluded that, a four-fold increase of recovery duration between serial SSG bouts did not affect the physiological, perceptual or time motion descriptors of experienced, and trained footballers during SSG bouts. The current results suggest that using a recovery duration ranging from 30 s to 120 s between serial SSG bouts will have a similar training effect for experienced and trained footballers. An implication of this finding, for coaches, is that planning and designing SSG with 30 s recovery durations separating the bouts will provide a more time efficient training session. Furthermore, coaches planning SSG training, may need to alter recovery durations outside of this range to increase the training stimulus. Lastly, experienced and trained footballers adopt pacing strategies during serial SSG sessions to maintain performance levels. Therefore, coaches should consider player experience, and exercise capacity when designing SSG training.

## Limitations

One limitation of this study is that pacing was not anticipated in the current design, and a subjective measure of pacing could have directly indicated if pacing was consciously used. Secondly, only one format of SSG was used, modifying the player numbers, pitch size, number of bouts and recovery periods, using goals and goalkeepers may have provided further insight of the effect of altering recovery duration on different SSG formats.

## Implications and Future Research

Implications of the current results for coaches planning SSG, is that the recovery duration of 30 s to 120 s does not affect physiological and perceptual responses, or speed and distance during the SSG bouts. However, future research should investigate how the technical component of SSG in experienced footballers is affected by increasing the recovery period from 30 s to 120 s.

## Supporting Information

S1 Data Raw data file. (XLSX)
